# Clinical practice guidelines for telesurgery, 2nd Edition: Committee for the Promotion of Remote Surgery Implementation, Japan Surgical Society

**DOI:** 10.1007/s00595-026-03295-z

**Published:** 2026-05-29

**Authors:** Masaki Mori, Satoshi Hirano, Kenichi Hakamada, Eiji Oki, Takayuki Ito, Shigeo Urushidani, Ichiro Uyama, Masatoshi Eto, Yuma Ebihara, Yukihide Kanemitsu, Kenji Kawashima, Takahiro Kanno, Masaru Kitsuregawa, Yusuke Kinugasa, Hiroaki Kitatsuji, Toshihiko Sato, Fumiaki Sato, Tomoki Shimokawa, Hiroshi Shimamoto, Shuji Takiguchi, Ichiro Takemasa, Masanori Tokunaga, Masaya Nakauchi, Hirokazu Noshiro, Masaki Mandai, Koshi Mimori, Hajime Morohashi, Tomoharu Yoshizumi, Go Watanabe, Yoshiharu Sakai, Norihiko Ikeda, Akinobu Taketomi

**Affiliations:** 1 Tokai University School of Medicine, 143 Shimokasuya, Isehara City, Kanagawa, Japan; 2 Department of Gastroenterological Surgery II, Division of Surgery, Faculty of Medicine, Graduate School of Medicine, Hokkaido University, Sapporo, Hokkaido Japan; 3 Department of Gastroenterological Surgery, Graduate School of Medicine, Hirosaki University, Hirosaki, Aomori, Japan; 4 Department of Advanced Medicine and Innovative Technology, Kyushu University Hospital, Fukuoka, Japan; 5 NHK Foundation, Tokyo, Japan; 6 Information Systems Architecture Science Research Division, National Institute of Informatics, Tokyo, Japan; 7 Department of Advanced Robotic and Endoscopic Surgery, Fujita Health University School of Medicine, Toyoake, Aichi Japan; 8 Department of Urology, Kyushu University Hospital, Fukuoka, Japan; 9 Department of Colorectal Surgery, National Cancer Center Hospital, Tokyo, Japan; 10 Graduate School of Information Science and Technology, The University of Tokyo, Tokyo, Japan; 11 RIVERFIELD Inc, Tokyo, Japan; 12 National Institute of Informatics, The University of Tokyo, Tokyo, Japan; 13 Department of Gastrointestinal Surgery, Graduate School of Medical and Dental Sciences, Institute of Science Tokyo, Tokyo, Japan; 14 Medicaroid Corporation, Hyogo, Japan; 15 Department of Thoracic, Breast and Pediatric Surgery, School of Medicine, Fukuoka University, Fukuoka, Japan; 16 A440 Inc, Tokyo, Japan; 17 Department of Cardiovascular Surgery, School of Medicine, Teikyo University, Tokyo, Japan; 18 Department of Gastroenterological Surgery, Graduate School of Medical Sciences, Nagoya City University, Aichi, Japan; 19 Osaka International Medical & Science Center, Osaka, Japan; 20 Department of General and Gastroenterological Surgery, Saga University, Saga, Japan; 21 Department of Gynecology and Obstetrics, Graduate School of Medicine, Kyoto University, Kyoto, Japan; 22 Department of Surgery, Kyushu University Beppu Hospital, Oita, Japan; 23 Department of Gastroenterological and General Surgery, Graduate School of Medical Sciences, Kyushu University, Fukuoka, Japan; 24 New Heart Watanabe International Hospital, Tokyo, Japan; 25 Osaka Red Cross Hospital, Osaka, Japan; 26 Department of Surgery, Tokyo Medical University, Tokyo, Japan; 27 Department of Gastroenterological Surgery I, Division of Surgery, Faculty of Medicine, Graduate School of Medicine, Hokkaido University, Sapporo, Hokkaido Japan; 28 Japan Surgical Society, Tokyo, Japan

**Keywords:** Remote surgery, Telesurgery, Telementoring, Surgical robots, Cybersecurity, Surgical education, Guidelines

## Abstract

Recent advances in surgical robotic systems, high-speed communication networks, and information processing technologies have made the clinical implementation of remote surgery increasingly feasible. Although pilot clinical applications have been initiated worldwide, the safe, ethical, and sustainable adoption of remote surgery requires comprehensive guidance that addresses not only technical considerations, but also clinical practice, legal responsibility, and organizational frameworks. In response to these needs, the Japan Surgical Society has developed the second edition of the Clinical Practice Guidelines for Telesurgery through a multidisciplinary, consensus-based process involving multiple surgical societies. This updated edition builds on validation and verification studies conducted since the publication of the first edition and places particular emphasis on practical implementation in real-world clinical settings, including telesurgical support and telementoring. The guidelines provide expanded, implementation-oriented recommendations covering surgeon and support staff qualifications, institutional requirements, communication network performance and cybersecurity standards, registry-based governance, and structured approaches to remote surgical mentoring. In addition, legal and ethical considerations are strengthened through the inclusion of representative informed consent documents and contractual frameworks. To enhance international applicability, content that is broadly relevant across jurisdictions is presented separately from elements specific to the Japanese regulatory environment. These guidelines aim to support the responsible global dissemination of telesurgery by promoting safety, transparency, and clinical effectiveness.

## Introduction

Advances in surgical robotic systems with expanded capabilities, the development of high-speed communication networks, and progress in information processing technologies have created a practical foundation for the clinical implementation of telesurgery [1, 2]. Telesurgery has the potential to improve access to surgical care in regions with limited medical resources, facilitate the dissemination of advanced surgical techniques, and expand educational opportunities in surgery, thereby contributing to improved healthcare delivery. Clinical trials and pilot implementations of telesurgery have already been initiated in Europe [3, 5], North America [4], Asia [6, 7], and Africa [8].

The safe implementation of telesurgery, however, requires careful consideration of multiple factors. Technical challenges inherent to remote environments, such as latency, network instability, and cybersecurity risks, must be adequately addressed through stable communication performance, reliable connectivity, and robust cybersecurity measures. In parallel, appropriate healthcare delivery frameworks are essential, including the selection of procedures suitable for telesurgery, clearly defined qualification standards for surgeons, surgical teams, and institutions, structured training programs, and comprehensive safety and emergency response protocols. Ethical and legal considerations are equally important, particularly with regard to the allocation of responsibility between local and remote surgeons and institutions, as well as the roles and responsibilities of robotic system manufacturers, telecommunications providers, and participating healthcare facilities. Furthermore, mechanisms to address communication costs and reimbursement of expenses incurred on the remote side are recommended to ensure financial sustainability.

In response to these challenges, the Japan Surgical Society published the Japanese version of the Clinical Practice Guidelines for Telesurgery in 2022 (https://jp.jssoc.or.jp/uploads/files/info/info20220622.pdf, in Japanese), followed by the English version in 2024 (9). In Japan, strong societal demand to reduce regional disparities in surgical care led to the early development of a legal framework for telesurgery in 2019. Subsequently, the Japan Surgical Society, in collaboration with related academic societies, robotic system manufacturers, and telecommunications providers, conducted a series of demonstration and validation studies aimed at the responsible clinical implementation of remote surgical support. The first edition of the guidelines was developed based on the outcomes of these studies and represented the first comprehensive guidelines dedicated specifically to telesurgery.

The second edition was developed by integrating the results of continued demonstration and validation studies conducted since the publication of the first edition [10–27].^1^ It identifies the practical challenges encountered in real clinical settings and provides more concrete, implementation-oriented guidance for future clinical trials and routine clinical adoption. In particular, this edition expands the guidance on telesurgical support by clarifying the qualification and competency requirements for surgeons and support staff, defining institutional requirements, specifying cybersecurity measures, and describing governance through registry-based management. In addition, recognizing that surgical telementoring is already in clinical use, this edition further addresses the requirements related to surgeons and participating institutions, network environments, equipment and devices, and program design, and includes representative examples of informed consent documents and contractual agreements.

The second edition of the Clinical Practice Guidelines for Telesurgery was developed through a consensus process involving the Japan Surgical Society and related academic societies. As an academically grounded and neutral set of guidelines, it is intended to support the safe, ethical, and responsible dissemination of telesurgery and to inform the development of appropriate regulatory and operational frameworks (https://jp.jssoc.or.jp/uploads/files/info/info20251211.pdf, in Japanese). In the English version, technical and legal principles that are broadly applicable across jurisdictions are presented first, followed by elements specific to the Japanese regulatory framework, while considering international applicability. The committee anticipates that these guidelines will contribute to the continued global advancement of telesurgery.

1. **Chapter 1 General remarks**
2. Aim
  The purpose of the clinical practice guidelines for telesurgery (hereinafter referred to as the Guidelines) is to present appropriate standards for the systems through which telesurgery is provided and implemented, specifically when performed remotely by a supervising physician in a distant location using both surgical robots and information and communication technology.

1.2. Types of telesurgery and the scope of the Guidelines [Table. 1]

1.2.1. Types of telesurgery
  In the Guidelines, telesurgery is classified into the following three types according to the degree of involvement of the remote physician in the surgery.

1.2.1.1. Telementoring
  The supervising physician remotely participates in real-time on-site surgery, with the patient present. This is a form of providing specific surgical guidance to the local surgeon through both images and voice using information and communication devices, such as tablets. It also includes the use of a system to remotely project lines and arrows on a local monitor and to supervise the entire operation by observing and understanding the physical environment of the local operating room.
  The relationship between a remote supervisor and a local surgeon corresponds to D to D (doctor-to-doctor) category defined in Japan’s Guidelines for the Appropriate Implementation of Online Clinical Practice (https://www.mhlw.go.jp/content/001126064.pdf (In Japanese), hereinafter referred to as the Guidelines for Online Clinical Practice), specifically pertaining to physician-to-physician information exchange and educational activities.

1.2.1.2. Telesurgical support
  The supervising physician operates the surgical robot remotely to assist the local surgical team as an assistant or partially as a surgeon, and performs the procedure collaboratively with the local surgeon. In the event of communication failure or other unforeseen circumstances, the local surgical team must be capable of completing the operation without the aid of the remote surgeon. The relationship between the remote supervising doctor, the patient, and the local doctor corresponds to D to P with D category in the Guidelines for Online Clinical Practice in Japan.

1.2.1.3. Full telesurgery
  In an environment in which the surgeon is not present at the local medical facility, a remote surgeon performs surgical procedures with complete remote control of the surgical robot. The relationship between the remote surgeon and the patient is classified as D to P category in the Guidelines for Online Clinical Practice. Although technically feasible, it has not been approved for implementation in Japan because of patient safety and ethical concerns Table 1.

**Table. 1:** Summary: Types of telesurgery

Types	Telementoring	Telesurgical support	Full telesurgery
Outline	A remote supervisor gives verbal and graphical instructions to local surgeons remotely using tablets and other information and communication devices	A remote supervisor remotely operates the surgical robot and directly assists the local surgical team as a surgeon or assistant	A telesurgeon operates on a patient directly with complete remote control in an environment where there is no surgeon on site
Robotic operator	Local surgeon (100%)	Local surgeon and remote surgeon (Collaborative surgery is achieved by switching operating authority.)	Remote surgeon (100%)
Primary responsibility	Local surgeon	Local surgeon	Remote surgeon
Prior confirmation of liability proration	Required	Required	Not required
Emergency response	Local surgeon	Local surgeon	Local staffs other than surgeon
Types of online clinical practice in Japan*	D to D	D to P with D	D to P
Legal feasibility	Yes	Yes	No at present in Japan

*D doctor, P patient*

1.2.2. Scope of the Guidelines
  These guidelines apply to all three types of remote surgery. They primarily target telesurgical support, in which a supervising surgeon performs direct surgical procedures remotely, provided that a surgeon capable of an emergency response is present locally. They also include telementoring, which is expected to become more widespread and requires certain conditions for real-time implementation. Full telesurgery is also included in these guidelines. However, due to numerous challenges regarding safety assurance in surgical techniques and patient management, as well as legal considerations, its implementation in Japan is currently difficult.

1.3 Definition of Terms
  The terms used in the Guidelines are defined as follows.

1.3.1. Surgical robot: A robotic surgical unit that has been approved as a surgical support system by an accredited agency. In Japan, it is described as an endoscopic surgical support device for medical reimbursement purposes.

1.3.2. Robot-assisted (endoscopic) surgery: Endoscopic surgery performed using a surgical robot. In Japan, it is described as endoscopic surgery using endoscopic surgical support devices under the medical reimbursement system.

1.3.3. Remote surgery or telesurgery (telesurgical support/telementoring/full telesurgery): Telepresence surgery, surgical assistance, and surgical guidance by a remote supervising surgeon at different health care facilities using information and communication technology and surgical robots.

1.3.4. Local hospital (institution): An actual facility where the patient undergoing surgery is located.

1.3.5. Local surgeon: A surgeon who is physically present at the same facility where the patient undergoing surgery is located.

1.3.6. Local operative staff: Personnel other than the local surgeon (e.g., physicians, anesthesiologists, clinical engineers, nurses, and medical information managers) at the facility where the patient undergoing surgery is located.

1.3.7. Remote hospital [institution]: A facility in which a physician in a supervisory role is present and provides remote surgical support and surgical guidance via information and communication technology, including images and audio, to the patient undergoing remote surgery.

1.3.8. Remote surgeon: A physician in a supervisory role who provides technical support to the local surgeon by operating a surgical robot from a remote facility.

1.3.9. Remote mentor: A physician who provides instruction using images and audio from a remote facility, but does not directly perform surgical procedures.

1.3.10. Remote operative staff: Personnel other than the remote surgeon/mentor (e.g., physicians, anesthesiologists, clinical engineers, nurses, and medical information managers) in a remote facility.

1.3.11. Facility Administrator (Administrator/Director of Hospital [Institute]): The head of the facility (hospital) who has management responsibilities for all medical services, including remote surgery, at the local facility or remote facility performing telesurgery.

1.4. How to use the Guidelines
  These guidelines are recommended for use as a reference when performing remote surgery in clinical settings. Although these guidelines cover content applicable across various medical specialties and organs, specific indications for telesurgery for various diseases and detailed surgical techniques should adhere to the guidelines established by the relevant academic societies specializing in those diseases.

2. **Chapter 2 Telesurgical support**

2.1. Provision framework for telesurgical support

2.1.1. Requirements for medical teams providing telesurgical support

2.1.1.1. Requirements for surgeons involved in telesurgical support

2.1.1.1.1. Remote surgeon
  A remote surgeon must possess sufficient technical skills to safely perform telesurgery while supporting a local surgeon. In Japan, remote surgeons must hold certification as a robot-assisted surgery instructor/proctor or an equivalent qualification from relevant societies in each field, such as the Japanese Society of Endoscopic Surgery or the Japan Robotic Surgery Society. Furthermore, the qualification must correspond to the specific surgical robot to be used. Additionally, they must complete the Training Program for Remote Surgery established and managed by the Japan Surgical Society in collaboration with relevant societies.

2.1.1.1.2. Local surgeon
  The local surgeon, as the primary operating surgeon, must complete the training program provided by the manufacturer of the surgical robot used and obtain a certificate as a robot-assisted endoscopic surgery operator from the relevant academic society in Japan. Furthermore, they must have completed the Training Program for Remote Surgery managed and operated by the academic society.
  The local surgeon must possess board certification in their respective field and have prior experience performing the planned procedure or have received direct instruction from an instructor/proctor certified by the relevant academic society at least once. However, if specific requirements are stipulated by the relevant academic society, then those requirements shall take precedence."

2.1.1.2. Requirements for operative staff involved in telesurgical support

2.1.1.2.1. Surgical assistant
  Physicians serving as surgical assistants at local facilities must complete the assistant training program provided by the manufacturer and distributor of the surgical robot being used. They must also have completed the “Training Program for Remote Surgery” managed and operated by the academic society in Japan.

2.1.1.2.2. Nurses
  For surgeries performed at the local facility, it is desirable that a nurse who has completed the training program provided by the manufacturer of the surgical robot, or who possesses equivalent or superior knowledge, participate directly as an assistant. However, the presence of a nurse at a remote facility is not mandatory. Furthermore, they must have completed the Training Program for Remote Surgery managed and operated by an academic society.

2.1.1.2.3. Clinical engineers
  Clinical engineers with experience in the maintenance and management of surgical robots must be assigned to both local and remote facilities. These clinical engineers are required to provide appropriate advice and troubleshooting for issues arising with the surgical robots, excluding those related to the communication environment. Clinical engineers at both the local and remote facilities must have completed the training program provided by the manufacturer of the surgical robot being used or possess knowledge equivalent to or exceeding that program. Furthermore, they must have completed the “Training Program for Remote Surgery” managed and operated by the academic society in Japan. Note that clinical engineers participating as scrub assistants must also possess the same knowledge and qualifications.

2.1.2. Requirements for medical facilities providing telesurgical support

2.1.2.1. Requirements for remote and local facilities providing telesurgical support

2.1.2.1.1. Local and remote facilities must have a medical safety management system as stipulated by law. In Japan, this corresponds to the Medical Care Act.

2.1.2.1.2. Local and remote facilities must establish information security management systems. In Japan, compliance with the latest version of the Guidelines on Safety Management of Healthcare Information Systems is required.

2.1.2.1.3. Local and remote facilities must have the necessary communication environment established for telesurgical support (see 2.1.3 Requirements for the communication network environment for performing telesurgical support).

2.1.2.1.4. Local and remote facilities must prepare a communication environment (such as a web conferencing system) that allows the remote surgeon to visually confirm the surgical environment at the local facility and engage in interactive voice communication.
  Furthermore, while vital signs, including the patient’s ECG and blood pressure, are monitored by the local surgeon and anesthesiologist, it is desirable to perform the procedure in an environment where the remote surgeon can continuously monitor this information. This communication environment must use a separate communication line system independent of the communication lines used by the remote surgery system.

2.1.2.1.5. Local and remote facilities must have a department (such as a Medical Information Department) responsible for maintaining and managing the information infrastructure, including the communication environment within the facility. These departments must be well-versed in the communication environment for telesurgical support, collaborate between facilities during the implementation of telesurgical support, and strive to maintain the communication environment.

2.1.2.2. Requirements for local facilities receiving telesurgical support

2.1.2.2.1. The local facility must meet the facility criteria for robot-assisted surgery under the medical fee reimbursement system for at least one surgical procedure and must have submitted notification of this to the Regional Bureau of Health and Welfare in Japan. However, the surgical procedures planned for telesurgical support do not need to be included in the notified procedures.
  Furthermore, if a relevant academic society establishes regulations regarding the implementation record of robot-assisted surgery at the local facility, those regulations shall take precedence.

2.1.2.2.2. Prior to providing telesurgical support, the local facility must have performed at least one case of the relevant robotic surgical procedure at its own facility. However, if a relevant academic society stipulates requirements regarding the facility’s track record of performing the procedure, those stipulations shall take precedence.

2.1.2.2.3. The local facility must possess the equipment and staff necessary to complete robotic surgical procedures planned for telesurgical support through alternative approaches, such as endoscopic surgery or open abdominal or thoracic surgery, in preparation for situations in which telesurgical support becomes difficult to continue.

2.1.3. Requirements for the communication network environment for telesurgical support

2.1.3.1. The communication network environment for telesurgical support must have the communication bandwidth required for stable operation of a surgical robot.
  It is desirable to utilize lines with guaranteed bandwidth or assured forwarding that ensure the required bandwidth. If a so-called best-effort type line with variable available bandwidth is used, it must be confirmed in advance that the communication bandwidth stably exceeds the bandwidth required for an operation in which a surgical robot is to be used. Note that the required bandwidth varies depending on the model of the surgical robot and the video compression method.

2.1.3.2. It is necessary to confirm in advance that the communication line does not have large delays, significant jitter, or packet loss. Note that with best-effort lines, the communication environment at the time of surgery may differ significantly from that at the time of prior confirmation, as delay fluctuations and packet loss can change significantly from instance to instance.

2.1.3.3. The sum of the round-trip communication network transmission delay time and information processing delay time,* newly generated in the telesurgery environment, should be within 100 ms (0.1 s) at the maximum.

*Information processing delay time: Time required to compress and decompress information signals for transmission and reception between remote locations.

2.1.3.4. The communication line for telesurgical support must be capable of implementing the security measures described in "2.1.6 Security measures necessary for telesurgical support" below.

2.1.3.5. To mitigate risks during communication failures (such as line disconnections or network congestion), it is desirable to implement redundant communication line configurations. Redundancy includes not only redundant communication line types but also redundant communication carriers. In such cases, it is necessary to confirm beforehand that surgical operations and the operation of surgical robots will not be affected during line disconnections, line switching, or line restoration.

2.1.4. Requirements for surgical robots and devices used in telesurgical support

2.1.4.1. The surgical robot used must be a medical device approved by an accredited organization. In Japan, the surgical robot must be a medical device officially approved as a highly controlled medical device (Class III) in accordance with the Act on Securing Quality, Efficacy, and Safety of Products Including Pharmaceuticals and Medical Devices and other relevant laws.

2.1.4.2. The surgical robot must be approved for use in telesurgical support.

2.1.4.3. The surgical robot must publicly disclose the communication bandwidth required for stable operation. If the required bandwidth fluctuates, the range must be specified.

2.1.4.4. The surgical robot must have specifications that assume and adjust for instantaneous communication breakdowns, communication delays and packet losses. Specifically, the robot must incorporate functions to mitigate control device stoppages or malfunctions when communication interruptions, delays, packet loss, or packet reordering occur.

2.1.4.5. Devices directly attached to and used with the surgical robot must be those recommended by the manufacturer and distributor of the robot being used from a safety perspective.

2.1.4.6. The surgeon’s console (cockpit) of the surgical robot must have the capability to switch operating privileges between the local and remote surgeons and to record the history of any such changes.

2.1.5. Information security management framework required for telesurgical support
  Surgeons, surgical staff, facility administrators at both local and remote sites, manufacturers and distributors of surgical robots, and telecommunications providers must implement information security measures in accordance with laws, regulations, and the latest guidelines to ensure the availability of telesurgical support.
  In Japan, compliance with the latest versions of the Guidelines for Online Clinical Practice, Guidelines on Safety Management of Healthcare Information Systems (https://www.mhlw.go.jp/content/10808000/001102570.pdf, in Japanese), and Safety Management Guidelines for Providers of Information Systems and Services that Handle Medical Information (https://www.soumu.go.jp/main_content/000891033.pdf, in Japanese) is required.

2.1.5.1. Surgeons and facility administrators at both local and remote facilities are required to have basic knowledge regarding the implementation of telemedicine. In Japan, they must complete government-designated Online Clinical Practice training courses. It is also desirable for surgical staff at both local and remote facilities to fully understand the content of the Guidelines for Online Clinical Practice.

2.1.5.2. Surgeons, surgical staff, and facility administrators at both local and remote facilities must independently implement information security measures at their facilities in accordance with the applicable laws, regulations, and guidelines.
  In Japan, compliance with the Guidelines for Online Clinical Practice and the Guidelines on Safety Management of Healthcare Information Systems is required.

2.1.5.3. Surgical robot manufacturers and telecommunication carriers that provide a telesurgical environment should establish a system that enables the safe implementation of telesurgical support in cooperation with local and remote facilities, based on laws, regulations, and guidelines.
  In Japan, compliance with the Safety Management Guidelines for Providers of Information Systems and Services that Handle Medical Information is required.

2.1.5.4. Contracts or agreements between local surgeons/facility administrators and remote surgeons/facility administrators regarding the implementation of telesurgical support must include a description of the information security management system, as indicated in “2.1.6. Security Measures Required for Telesurgical Support.”

2.1.6. Security measures required for telesurgical support
  The integrity and confidentiality of information security must be guaranteed while ensuring the availability of telesurgical support. Specifically, local and remote facilities must implement appropriate measures for all aspects related to maintaining the telesurgical support environment. These include equipment-related, technical, organizational (such as instructions and accountability systems), and personnel (such as education and training) measures.

2.1.6.1. Security measures for telecommunication lines
  Security measures must be implemented from three perspectives: inter-facility connections, connections for surgical equipment maintenance and management, and intra-facility connections.

2.1.6.1.1. A closed communication network, such as a layer-3/layer-2 virtual private network (L3/L2VPN), that is physically or logically separated from the Internet is desirable. If it is unavoidable to use partially open lines, highly secure methods, such as IPSec+IKE (version 2) connections, are recommended. At the same time, it is necessary to limit the destination/source IP addresses and port numbers used as much as possible by means of firewalls.

2.1.6.1.2. When performing maintenance of surgical robots remotely from outside, the company entrusted with maintenance work must prepare a maintenance management plan detailing security compliance items, such as access methods and authority management, depending on the scope of the maintenance tasks. This plan must define the handling of administrative privileges, rules, and procedures for accessing the system, and obtain facility approval.

2.1.6.1.3. When using VPN devices, it is necessary to keep the firmware and other components up to date to ensure that they are free of vulnerabilities. It is also important to note that the risk of IP address spoofing is not zero.

2.1.6.1.4. Remote surgical environments and medical information system networks, such as electronic medical record systems, must avoid intra-facility connections and be physically or logically isolated from other lines.

2.1.6.2. Security measures for surgical robots (including information processing terminals)

2.1.6.2.1. When using communication lines other than a closed communication network, communication encryption devices must be installed between the surgical robots at local and remote facilities, or between the information processing terminals connected to the communication lines at both facilities.

2.1.6.2.2. Vulnerability information on the OS and middleware in the control system of the surgical robot must be constantly checked. If a vulnerability is found, appropriate measures, such as applying a correction program (patch), must be taken as soon as possible according to the severity of the vulnerability.

2.1.6.2.3. It is necessary to clarify the setting of accessing privileges to the robot system, such as privilege IDs with access rights to set all parameters for the surgical robot, operation administrator IDs, robot operator IDs, etc., and to authenticate and authorize access to the minimum number of personnel deemed necessary for business purposes.

2.1.6.2.4. When connecting information and communication devices, such as PCs, or storage media, such as USB drives, to a remote surgical environment, it is necessary to check them beforehand using malware countermeasures software.

2.2. Implementation framework for telesurgical support

2.2.1. Preparatory procedures at the facility for the implementation of telesurgical support
  To implement telesurgical support, the local and remote facilities must establish both the provision and implementation frameworks outlined in these guidelines. These frameworks must be approved by the facility’s safety management system (e.g., the Medical Safety Management Committee).
  In Japan, both local and remote facilities are required to obtain institutional approval of this technology as a Highly Difficult New Medical Technology, as stipulated in the Medical Service Act, before commencing telesurgical support. The measures required for using Highly Difficult New Medical Technology are obligatory for approval as an Advanced Treatment Hospital from the government. Hospitals other than those designated as Advanced Treatment Hospitals may delegate the review to an external committee for the evaluation of Highly Difficult New Medical Technology.
  Facilities implementing telesurgical support must participate in a registry system established by an organization (provisional name: Remote Surgery Management Organization) comprising interdisciplinary societies involved in remote surgery and relevant societies in each field. and must report their performance.

2.2.2. Surgical procedures for which telesurgical support can be provided
  Surgical procedures that can be performed with telesurgical support are based on regulations and guidelines from government and academic societies.
  In Japan, robotic surgery procedures covered by insurance as surgery using endoscopic surgical support devices are generally allowed to be performed as telesurgical support. However, it should be noted that surgical procedures that are accepted as covered by insurance differ depending on the surgical robot model.
  Furthermore, with regard to feasible surgical procedures, the difficulty and safety of procedures vary significantly by specialty, and the circumstances for implementing remote surgical support also differ. Therefore, all procedures must be performed in strict compliance with the regulations established by each relevant academic society. Note that the surgical procedures specified in each society’s regulations require approval by an organization (provisional name: Remote Surgery Management Organization) comprising interdisciplinary societies involved in remote surgery and relevant societies in each field."

2.2.3. Preparation for implementing telesurgical support

2.2.3.1. Education of local surgeons and surgical staff
  It is recommended that local surgeons and surgical staff observe actual telesurgical support at other facilities and use the experience to prepare for implementation at their own facilities. Local staff involved in telesurgical support should prepare an operation manual, hold regular conferences and study groups, and establish an adequate education system for staff. The manual must be shared among local and remote facility staff when telesurgical support is provided. The materials and educational programs must also be reviewed as appropriate. (Reference Material: Telesurgical Support Operational Manual). If relevant academic societies in each field have created and approved materials, those shall be used.

2.2.3.2. Preoperative review of cases
  The local surgeon, local surgical staff, and remote surgeon must hold a conference in advance to thoroughly discuss the appropriateness of providing telesurgical support (patient condition, indication, surgical procedure, etc.), as well as the division of roles between the local and remote surgeons, the possibility of changing the surgical procedure, and what to do if it becomes difficult to perform the procedure as telesurgery. The details of these discussions must be documented in the patient’s medical record. Regarding the extent of remote surgical assistance, it is necessary to confirm that adjustments are made as appropriate during surgery to ensure patient safety and that the remote surgeon may take the lead when necessary.
  When using a web conference system, the patient ‘s consent must be obtained in advance regarding the handling of the patient’s personal information, and appropriate security management in compliance with relevant guidelines must be implemented."

2.2.3.3. Preoperative explanation to patients and obtaining informed consent
  When obtaining consent from a patient, the local surgeon or a physician at the local surgical site must provide the patient with an overview of telesurgical support, information about the remote surgeon, the advantages of telesurgical support, and possible disadvantages, in addition to general preoperative explanations. The following explanation must also be included.

2.2.3.3.1. During telesurgical support, the remote surgeon performs certain surgical procedures using a surgical robot at a remote facility.

2.2.3.3.2. The patient’s personal information and disease status are provided to the remote surgeon.

2.2.3.3.3. Even in the event of a malfunction of the information and communication devices and other devices between the surgical robots, the local surgeon and surgical staff can safely continue the ongoing surgery.

2.2.3.3.4. The local and remote facilities must take sufficient security measures to prevent unauthorized access by third parties and information leakage.

2.2.3.4. Pre-operational verification of communication network and implementation environment
  Local and remote facilities must have a provision and implementation framework for remote surgical support, as outlined in these guidelines, approved in advance by the facility’s safety management system (such as the Medical Safety Management Committee).
  Local and remote surgical staff must use a checklist to verify the communication and implementation environments between both facilities in advance. The checklist must include the operational status of the surgical robots and devices and the communication status of the video, audio, and patient monitors between the local and remote facilities. (Reference Material: Telesurgical Support Operational Manual Checklist).
  In fields where operational manuals, emergency response manuals, and checklists have been created and approved by relevant academic societies, these documents should be used.

2.2.4. Ensuring safety during telesurgical support
  To prepare for emergencies during telesurgical support, a means of emergency communication (e.g., mobile phones) between operating rooms at local and remote facilities must be confirmed in advance.
  When implementing telesurgical support, both the local and remote surgical teams must include clinical engineers proficient in the maintenance and management of the surgical robot being used. Furthermore, a system must be established to ensure emergency contact with the responsible personnel of the surgical robot manufacturer.
  Information regarding the implementation date and time of telesurgical support must be shared with the department responsible for maintaining the facility’s communication infrastructure (e.g., the Medical Information Department). A system must be in place to promptly notify this department if communication line abnormalities occur during surgical support. In addition, it is desirable to have a system in place that allows for urgent contact with the telecommunications provider supplying the lines.

2.2.5. Response to adverse events during telesurgical support
  Local/remote surgeons and local surgical staff should prepare and share an Emergency Response Manual in advance to address potential intraoperative emergencies. If a situation arises during telesurgical support that makes it impossible to perform the surgery properly or to ensure patient safety, the local surgeon or local surgical staff must take the initiative to promptly decide whether to suspend or terminate the telesurgery and proceed with actions according to the Emergency Response Manual. The manual must include the criteria for the decision to suspend or discontinue telesurgical support, as well as the criteria for conversion to open or laparoscopic surgery. This manual must also be reviewed regularly. (Reference Material: Telesurgical Support Emergency Response Manual)

2.2.6. Change in a planned surgical procedure during telesurgical support
  During telesurgical support, if the local and remote surgeons determine through consultation that a change in the surgical procedure is necessary, telesurgical support may continue provided that the modified procedure is permitted under the guidelines and the procedure can be completed at the local facility, even if telesurgical support becomes difficult.

2.3. Apportionment of responsibility for telesurgical support
  In principle, the local surgeon and local facility administrator shall bear responsibility for outcomes related to telesurgical support, including medical procedures and postoperative complications. Furthermore, the local surgeon and local facility administrator must thoroughly discuss with the remote surgeon and remote facility administrator for each case whether responsibility will be shared (and if so, the details and extent of such sharing) and create a written agreement or equivalent record in advance.

2.4. Patient-doctor relationship in telesurgical support
  The local surgeon must treat the patient directly as the attending physician or equivalent. In contrast, the remote surgeon may participate in telesurgical support without having been involved in prior direct patient care; however, it is desirable for the remote surgeon to arrange an opportunity to consult with the patient using a web conferencing system or similar means. In Japan, telesurgical support is classified as D to P with D category under the Guidelines for Online Clinical Practice.

2.5. Cost sharing in telesurgical support
  Telesurgical support ensures standard or above-standard quality of care with the participation of a remote surgeon with specialized skills.
  In Japan, the inclusion and treatment of telesurgical support in the reimbursement system have not yet been determined; however, but it should be evaluated at least the same or better than the same procedure performed in conventional robot-assisted surgery.
  In addition to the surgical costs incurred locally, telesurgical support incurs supplemental costs for personnel, facilities, and equipment at the remote facility where surgical support is provided, as well as communication line costs. In particular, the fees for guaranteed bandwidth lines with a high level of communication security are expensive at present, and the use of more economical lines and methods to bear high line costs should be addressed in the future.
  To expand the number of sites supported by telesurgery, it is desirable to use virtual leased circuits that allow multipoint connections. It is also necessary to promote the emergence of economical communication services that guarantee the minimum bandwidth required for the operation of surgical support robots. Considering that telesurgical support is essentially operated from a humanitarian perspective, targeting elderly patients living in remote areas who have difficulty moving, it is important to make it eligible for public subsidies.

3. **Chapter 3 Telementoring**
  In these guidelines, telementoring is defined as the use of images and audio by a telesurgical instructor to guide a local surgeon in real time, regardless of the surgical procedure, for example, robot-assisted, thoraco/laparoscopic, or open chest/abdominal surgery.

3.1. Provision Framework for telementoring

3.1.1. Requirements for surgeons involved in telementoring

3.1.1.1. Remote mentor
  Remote mentors must possess sufficient technical skills to safely guide local surgeons during telementoring. For remote instruction in endoscopic and robot-assisted surgery, remote mentors must hold relevant qualifications from the respective specialty societies (e.g., a certified surgeon or proctor).

3.1.1.2. Local surgeon
  No specific requirements are specified.

3.1.2. Requirements for medical facilities providing telementoring
  The local facility must prepare a communication environment that enables the remote mentor to visually monitor the surgical environment and communicate via audio. While the local surgeon, anesthesiologist, and other personnel are responsible for monitoring the patient’s vital signs, including the electrocardiogram and blood pressure, it is desirable to perform the procedure in an environment in which the remote mentor can continuously review this information.

3.1.3. Requirements for a communication network environment to provide telementoring

3.1.3.1. The communication environment used for telementoring varies greatly depending on the amount of video information to be transmitted, the required stability and completeness of communication, real-time performance, and economic efficiency. Therefore, it is necessary to construct and implement an appropriate communication environment according to the specific demands of telementoring.

3.1.3.2. Open lines with an Internet connection, or so-called best-effort lines, are lines where communication delay, delay fluctuation, and packet loss can change from moment to moment. It should be noted that the communication environment during actual telementoring may differ significantly from that in prior confirmation.

3.1.3.3. A closed communication network that is physically or logically isolated from the Internet (Virtual Private Network (Layer-3/Layer-2 VPN)) is desirable but not mandatory. When using an Internet connection with VPN devices at both ends, it is desirable to employ a highly secure method such as IPSec+IKE (version 2) connections, and to restrict destination/source IP addresses and used port numbers via firewalls or similar measures.

3.1.3.4. The communication lines used for telementoring must be physically or logically separated from hospital information system, such as an electronic medical record system.

3.1.3.5. Alternative means of communication (such as telephone or mobile lines) must be secured in case of communication failure.

3.1.4. Requirements for equipment and devices used in telementoring

3.1.4.1. The device used for telementoring should ideally be medical equipment developed specifically for telementoring. In Japan, such a device should be approved as a Class II or higher-grade managed medical device under laws and regulations, such as the Act on Securing Quality, Efficacy, and Safety of Products Including Pharmaceuticals and Medical Devices, etc.

3.1.4.2. The device on the local surgeon’s side should ideally be capable of outputting not only the surgical field image during the procedure, but also video and audio of the entire operating room. It must also include output ports for monitors to view feedback video from the remote mentor and for speakers that enable the local surgeon to hear audio from the remote mentor.

3.1.4.3. The device on the remote mentor’s side must be capable of accurately sharing surgical field images from the local surgeon’s side and must include inputs such as cameras and microphones, as well as speaker output. Furthermore, it is desirable to include a touchscreen monitor that enables advanced information sharing, such as annotation drawing.

3.1.4.4. Accessories for connecting information terminals to medical devices, such as endoscopic cameras, as well as devices used for audio input/output, must be those recommended by the manufacturer and distributor of the system.

3.1.4.5. The resolution of the displays used (the surgeon’s monitor and the monitor attached to the remote physician’s terminal) should be at least full high-definition (FHD: 1920×1080 pixels) or higher.

3.1.5. Requirements for programs necessary for telementoring

3.1.5.1. The program used for image annotation by remote mentors should ideally be developed specifically for telementoring. In Japan, it is desirable that it be registered as a Class II or higher-managed medical device.

3.1.5.2. To determine whether the bandwidth of the communication line is suitable for performing telemontoring, it is desirable that communication delays be evaluated beforehand or that the system possess a function capable of displaying real-time delay measurement results and the communication bandwidth used during transmission.

3.1.5.3. Telementoring devices must feature a user interface and operability designed to allow both the local surgeon and remote mentor to simultaneously monitor the progress of the surgery, ensuring that no discrepancies arise in their conversation.

3.1.5.4. The video and audio quality for telementoring should ideally meet the following requirements.

Video

- Resolution: Full HD (FHD: 1920×1080 pixels) or higher
- Frame rate: 60fps or higher

Audio

- Sampling rate of 44.1 kHz or higher (equivalent to CD quality)
- Supports full duplex communication
- Equipped with echo cancellation functionality

3.1.6. Communication delay in telementoring
  In telementoring, significant perceived latency can hinder communication between the mentor and the surgeon. Perceived latency is the total latency time, which is the sum of the delay from the monitor and other equipment used, information processing (software) delay, and communication (network) delay. While telesurgical support recommends a combined communication and information processing delay of 100 milliseconds (2.1.3.3.), telementoring does not require strict delay time management. However, it is necessary to evaluate the perceived delay in advance. As a reference, the Society of American Gastrointestinal and Endoscopic Surgeons (SAGES) recommends a total delay time of ≤ 450 ms for telementoring [28].

3.1.7. Information Security Measures Required for Telementoring
  When handling patient personal identification information during telementoring, remote mentors, local surgeons, local surgical staff, staff, and local facility administrators must implement appropriate information security measures. In Japan, compliance with the latest version of the Guidelines on Safety Management of Healthcare Information Systems and the information security policies of both local and remote facilities is required.

3.1.7.1. Contractors providing telementoring environments that include patient identifiable information related to medical care must implement appropriate information security measures. In Japan, they must comply with the Guidelines for Security Management for Providers of Information Systems and Services Handling Medical Information, as well as the information security policies of both the local and remote facilities.

3.1.7.2. When telementoring is provided as part of telesurgical support or when surgical guidance requires high real-time responsiveness, information security measures equivalent to those for telesurgical support must be implemented.

3.1.7.3. In addition to not handling patients’ personally identifiable information, information security measures must be implemented to ensure that operating room footage does not contain staff personal information.

3.1.7.4. Advanced authentication and authorization management, such as biometric and multifactor authentication, shall be implemented separately for users and maintenance personnel of the remote surgical guidance system.
  The number of personnel shall be limited to the minimum necessary for operations, and log management of actual usage is required.

3.1.7.5. When a remote supervising physician uses a remote surgical guidance system via information and communication equipment not managed by their affiliated medical institution, the conditions permitting the use of the terminal (BYOD: Bring Your Own Device), the scope of use, management methods, OS updates, and other related matters must be included in the relevant regulations.

3.2. Implementation framework for telementoring

3.2.1. Surgical procedures for which telementoring can be provided.
  All robotic procedures can be performed under telementoring. In Japan, surgeries covered by national insurance are eligible for telementoring.

3.2.2. Preparation for implementation of telementoring

3.2.2.1. Preoperative review of cases
  The local surgeon and remote mentor must thoroughly review the details of the telementoring for the patient’s surgery and document them in the local facility’s medical records. The patient’s consent regarding the handling of their personal information must be obtained prior to the review with the remote mentor.

3.2.2.2. Preoperative explanation to patients and obtaining informed consent
  When obtaining patient consent, the local surgeon or physician staff member must explain to the patient, in addition to the general preoperative explanation, the outline of the telementoring, information about the remote mentor, the benefits of telementoring, and potential disadvantages that may arise (Telementoring Consent Form Template).

3.2.2.3. Pre-operational verification of communication network and implementation environment
  The local surgeon, local surgical staff, remote mentor, medical engineer managing equipment, and communications administrator must verify the communication environment and the operational status of the devices to be used between both facilities in advance.

3.2.2.4. Management of information and communication devices
  Regular updates and patches for the operating systems of information and communication devices used for telementoring, as well as vulnerability information, must be constantly monitored for availability from the manufacturer. If necessary, updates must be promptly implemented before conducting telementoring.

3.2.3. Response to adverse events during telementoring
  If a situation arises in which surgery cannot be performed appropriately or in which ensuring patient safety becomes difficult during telementoring, the local surgeon or local surgical staff must promptly determine whether to suspend or terminate the telementoring.

3.3. Apportionment of responsibility for telementoring
  In principle, the local surgeon and facility administrator are responsible for medical practices and results related to telementoring. However, specific details should be thoroughly discussed beforehand, and a written agreement or alternative record should be prepared. (Telementoring Consent Form Template).

3.4. Patient-doctor relationship in telementoring
  A remote mentor may provide telementoring without providing medical care to the patient. In Japan, telementoring is categorized as a form of D to D in the Guidelines for Online Clinical Practice.

**Fig. 1:**
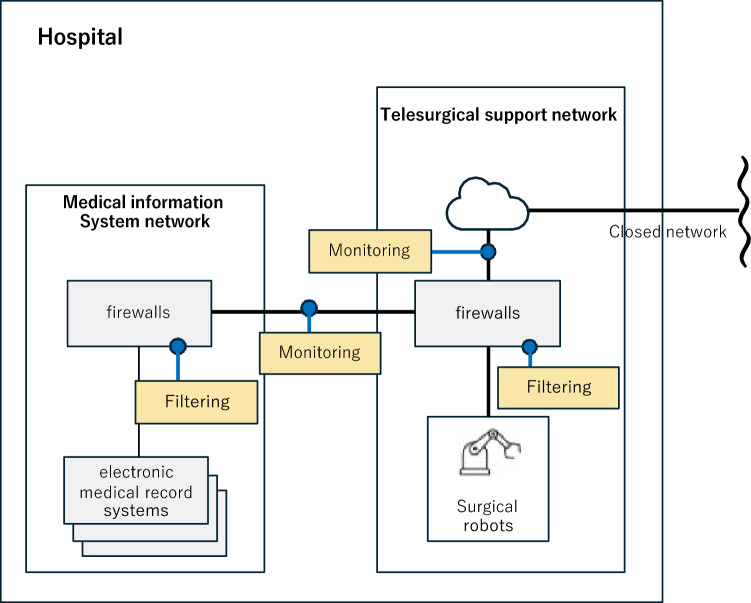
Intra-facility connections and security countermeasures

**Fig. 2:**
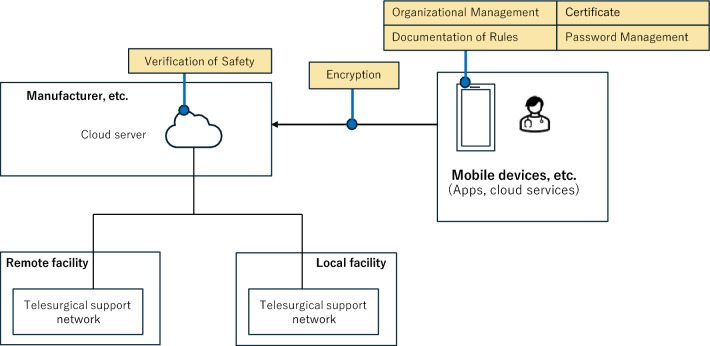
Cloud service connections and security countermeasures

**Fig. 3:**
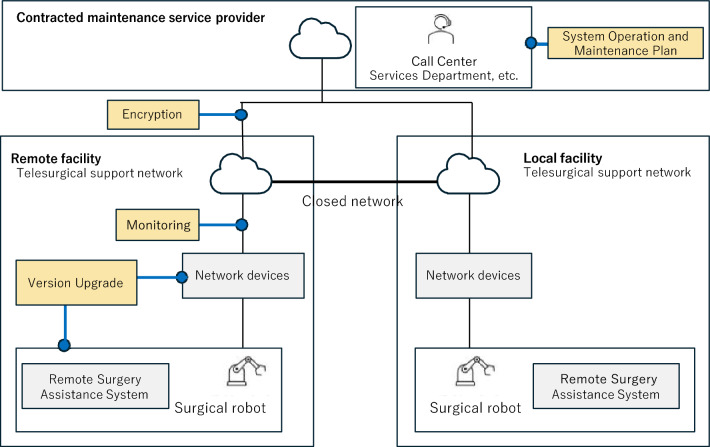
System maintenance and management connections and security countermeasures

4. **Chapter 4 Full telesurgery**
  Full telesurgery is designed for situations in which no surgeon capable of performing the procedure is present at the local facility, with the remote surgeon performing all surgical maneuvers using a surgical robot from a remote location. Therefore, it differs significantly from telesurgical support, in which local surgeons and surgical staff can continue the procedure if telesurgery is interrupted. Consequently, its implementation must guarantee extremely high reliability and safety.
  Furthermore, in Japan, as no interpretation has been provided regarding the relationship between full telesurgery and the prohibition of non-examination treatment under Article 20 of the Medical Practitioners Act, it is necessary for full telesurgery to be stipulated in the Guidelines for Online Clinical Practice as a condition for implementation.
  Due to numerous challenges, including legal issues, as well as technical and ethical considerations, implementing full telesurgery is currently difficult in Japan. However, with the innovative progress of surgical robots and information and communication technology, its future implementation in society is anticipated.

5. **Chapter 5 Comprehensive security measures**
  These guidelines assume a configuration that enhances the closed nature of both the remote surgery environment and the medical information system. This is achieved by avoiding direct internal connections between the remote surgery environment and the medical information system and by using secure networks, such as private communication networks between the two facilities and between surgical robot providers. This approach addresses both the need to ensure the safety of remote surgery and that of the hospital medical information system.
  However, as the methods of utilizing remote surgery evolve in the future, connections within the facility to medical information systems and other systems, as well as diverse external connections to parties other than the surgical robot provider, are also anticipated. In such cases, there is a risk of serious security incidents occurring, thus necessitating careful consideration when establishing comprehensive information security measures.
  Furthermore, when implementing security monitoring for a remote surgical environment, several factors must be considered: the monitoring equipment must be capable of recognizing the communications necessary to operate the surgical robot, and to enable efficient monitoring of multiple networks, the monitoring equipment should be physically located close proximity to the respective network devices [Figs. 1, 2 and 3].

5.1. Security measures for intra-facility connections

5.1.1. When connecting remote surgical environments to medical information system networks, the medical information system network shall also use firewalls, etc., to restrict destination/source IP addresses and port numbers as much as possible.

5.1.2. When connecting remote surgical environments to medical information system networks, information security measures, such as monitoring for unauthorized access, must be implemented between the medical information system network and the remote surgical support network.

5.1.3. Information security measures, such as monitoring for unauthorized access, must be implemented between the remote surgical support network connecting local and remote facilities.

5.2. Security measures for using cloud services

5.2.1. When utilizing a cloud service provided by a surgical robot manufacturer or distributor that allows verification of surgical operation histories performed using their surgical robots, it is necessary to confirm the security management of medical information based on the risk assessment of that service.
  Specifically, this includes verifying whether the cloud infrastructure used has third-party evaluations or certifications, such as ISMAP registration, assessing access management within the service (including privileged access management, identity and authentication management, physical security, etc.), reviewing management processes for system development and changes (development management, change management), system operation management (vulnerability management, incident management, system operation monitoring, network management, monitoring for deviations in various settings within cloud services, ensuring redundancy, etc.). If these are outsourced, then it is necessary to confirm the security management of medical information, including understanding the management status at the outsourcing provider.

5.2.2. For mobile devices and other information and communications equipment not managed by medical institutions that access cloud services (e.g., BYOD: Bring Your Own Device, devices using personally owned information equipment), it is necessary to include provisions in regulations regarding the conditions for permitting their use, the scope of use, management methods, and other related matters. Furthermore, for information and communications equipment used based on these provisions, including the status of usage permission, it is necessary to implement ledger management and other measures, similar to information and communications equipment managed by medical institutions.

5.2.3. When connecting to cloud services, encrypted communication must be used.

5.2.4. When accessing cloud services, it is necessary to identify and authenticate users and document procedures for user authentication methods and account management in rules, manuals, or similar documents.

5.2.5. When storing information containing personal data in cloud services, it is necessary to implement client authentication on the connected information and communications equipment.

5.2.6. When using passwords for user identification and authentication, it is necessary to set passwords that are secure and difficult for third parties to guess.

5.3. Security measures for system maintenance and management connections

5.3.1. When connecting to external lines for purposes such as robot maintenance and management, it is necessary to encrypt communications or information depending on the communication network used.

5.3.2. Depending on the content of maintenance and management operations, the contracted maintenance company must create a maintenance management plan. This plan must specify the information required for provision (such as the status of equipment like surgical robots, records of operation history and operating time, etc.), access methods, and security compliance items, such as permission management. The plan must define the handling of administrator permissions, rules, and procedures for accessing the system, and obtain approval from the facility.

5.3.3. When using VPN devices, ensure that the firmware and other components are kept up to date to maintain a state free of vulnerabilities. Regarding IP addresses, it is also necessary to be aware that the risk of impersonation is not zero.

5.3.4. Information security measures, such as monitoring for unauthorized access, must be implemented between the contracted maintenance company and the remote surgical environment.

Committee for the Promotion of Remote Surgery Implementation, Japan Surgical Society.

Committee members:

- Masaki Mori(Tokai University School of Medicine)
- Satoshi Hirano(Department of Gastroenterological Surgery II, Division of Surgery, Faculty of Medicine, Graduate School of Medicine, Hokkaido University)
- Kenichi Hakamada(Department of Gastroenterological Surgery, Graduate School of Medicine, Hirosaki University)
- Eiji Oki(Department of Advanced Medicine and Innovative Technology, Kyushu University Hospital)
- Takayuki Ito(NHK Foundation)
- Shigeo Urushidani(National Institute of Informatics, Information Systems Architecture Science Research Division)
- Ichiro Uyama(Department of Advanced Robotic and Endoscopic Surgery, Fujita Health University School of Medicine)
- Masatoshi Eto(Department of Urology, Kyushu University Hospital)
- Yuma Ebihara(Department of Gastroenterological Surgery II, Division of Surgery, Faculty of Medicine, Graduate School of Medicine, Hokkaido University)
- Yukihide Kanemitsu(Department of Colorectal Surgery, National Cancer Center Hospital)
- Kenji Kawashima(Graduate School of Information Science and Technology, The University of Tokyo)
- Takahiro Kanno(RIVERFIELD Inc.)
- Masaaru Kitsuregawa(National Institute of Informatics, The University of Tokyo)
- Yusuke Kinugasa(Department of Gastrointestinal Surgery, Graduate School of Medical and Dental Sciences, Institute of Science Tokyo)
- Hiroaki Kitatsuji(Medicaroid Corporation)
- Toshihiko Sato(Department of Thoracic, Breast and Pediatric Surgery, School of Medicine, Fukuoka University)
- Fumiaki Sato(A440 Inc.)
- Tomoki Shimokawa(Department of Cardiovascular Surgery, School of Medicine, Teikyo University)
- Hiroshi Shimamoto(NHK Foundation)
- Shuji Takiguchi(Department of Gastroenterological Surgery, Graduate School of Medical Sciences, Nagoya City University)
- Ichiro Takemasa(Osaka International Medical & Science Center)
- Masanori Tokunaga(Department of Gastrointestinal Surgery, Graduate School of Medical and Dental Sciences, Institute of Science Tokyo)
- Masaya Nakauchi(Department of Advanced Robotic and Endoscopic Surgery, Fujita Health University School of Medicine)
- Hirokazu Noshiro(Department of General and Gastroenterological Surgery, Saga University)
- Masaki Mandai(Department of Gynecology and Obstetrics, Graduate School of Medicine, Kyoto University)
- Koshi Mimori(Department of Surgery, Kyushu University Beppu Hospital)
- Hajime Morohashi(Department of Gastroenterological Surgery, Graduate School of Medicine, Hirosaki University)
- Konosuke Moritani(Department of Colorectal Surgery, National Cancer Center Hospital)
- Tomoharu Yoshizumi(Department of Gastroenterological and General Surgery, Graduate School of Medical Sciences, Kyushu University)

Advisors:

- Ryuichi Yamamoto(Medical Information System Development Center)
- Takafumi Ochiai(Atsumi & Sakai)
- Atsushi Kajitani(Kajitani Law Office)

Supervisors:

- Akinobu Taketomi(Japan Surgical Society/Department of Gastroenterological Surgery I, Hokkaido University)
- Norihiko Ikeda(Japan Surgical Society/Department of Surgery, Tokyo Medical University)
- Yoshiharu Sakai(Japan Society for Endoscopic Surgery/Osaka Red Cross Hospital)
- Go Watanabe(Japan Robotic Surgery Society/New Heart Watanabe International Hospital)

Collaborators:

- Takeshi Inazawa(Ryobi Systems Co., Ltd.)

This second edition of the Guidelines was created by consolidating the opinions of the following academic societies and reaching a consensus:

- Japan Surgical Society
- The Japanese Society of Gastroenterological Surgery
- The Japanese Association for Chest Surgery
- The Japanese Society for Cardiovascular Surgery
- Japanese Society of Pediatric Surgeons
- Japan Association of Endocrine Surgery
- Japanese Society of Otorhinolaryngology Head and Neck Surgery
- The Japanese Urological Association
- Robot-Assisted Cardiac Surgery Council
- Japan Robotic Surgery Society
- Japanese Society of Hepato-Biliary-Pancreatic Surgery
- The Japanese Association for Thoracic Surgery
- Japan Society of Obstetrics and Gynecology
- Japan Society of Gynecologic and Obstetric Endoscopy and Minimally Invasive Therapy
- Japan Society for Endoscopic Surgery
- Japanese Society of Endourology and Robotics

These guidelines were prepared by collecting public comments from the following societies:

- Japan Surgical Society
- The Japanese Society of Gastroenterological Surgery
- The Japanese Association for Chest Surgery
- The Japanese Society for Cardiovascular Surgery
- The Japanese Society of Pediatric Surgeons
- Japan Association of Endocrine Surgery
- Japan Robotic Surgery Society
- Japanese Gastric Cancer Association
- Japan Telemedicine Society
- Japanese Society of Hepato-Biliary-Pancreatic Surgery
- Japanese Ophthalmological Society
- The Japanese Association for Thoracic Surgery
- Japan Society of Obstetrics and Gynecology
- Japan periOperative Nursing Academy
- Japan Esophageal Society
- The Japanese Orthopaedic Association
- The Japan Society of Coloproctology
- Japan Society for Endoscopic Surgery
- Japanese Breast Cancer Society
- The Japan Neurosurgical Society
- Japanese Society of Endourology and Robotics
- Japanese Society of Anesthesiologists
- Japan Association for Clinical Engineers

## Footnotes

1 The articles (10)-(27) are the results obtained from the "Demonstration Study for Establishment of Guidelines for Remote Surgery Using Surgical Robots" in the "Advanced Telemedicine Network Research Project" of the Japan Agency for Medical Research and Development (AMED) since FY2019, and "Demonstration Research for Implementing Remote Surgery Using Surgical Robots” in the "Advanced Telemedicine Network Practical Application Research Project" (AMED) since FY2022.
